# Estimating the reliability of round‐robin judgments with social relations confirmatory factor analyses

**DOI:** 10.1111/bmsp.70043

**Published:** 2026-03-25

**Authors:** Steffen Nestler, Oliver Lüdtke, Alexander Robitzsch

**Affiliations:** ^1^ University of Münster Münster Germany; ^2^ Leibniz Institute for Science and Mathematics Education Kiel Kiel Germany

**Keywords:** confirmatory factor analysis, reliability, social relations model, structural equation modelling

## Abstract

The social relations model (SRM) is commonly used in psychological research to analyse interdependent data from round‐robin designs, where all members of a group rate each other. Based on the recently suggested social relations confirmatory factor analysis (SR‐CFA), we present general formulas for determining the reliability of composites of round‐robin judgments and also derive simpler variants when specific restrictions are applied to the parameters of the SR‐CFA model. In the unidimensional case, this results in an omega‐type reliability measure, which can be converted into an alpha‐type reliability measure through further restrictions. We also discuss how standard errors of the reliability coefficients can be obtained, illustrate the suggested methods using an empirical example, and we examine the suitability of the estimation approach in a small simulation study. Finally, we discuss questions for future methodological research and how the person‐level composites are related to other SRM effect estimates proposed in the SRM literature.

## 1 INTRODUCTION

A great deal of psychological research examines the causes and consequences of interpersonal perception and judgment. In educational psychology, for example, researchers are interested in the performance of pupils in cooperative learning groups (e.g., Horn et al., 1998). Similarly, social and personality psychologists study interpersonal attraction between unacquainted individuals (e.g., Küfner et al., 2012) or the accuracy and bias in self‐ and other‐perception (e.g., Kwan et al., 2004). Finally, clinical psychologists seek to understand patient's perceptions in group psychotherapy (e.g., Christensen & Feeney, 2016).

The typical design used to investigate such questions is the round‐robin design in which all members of a group interact with one another, or each respondent reports on their perceptions of the other group members. For example, each group member is asked to judge how nervous each of the other group members is or how much they like each of the other group members. The resulting interdependent data are then analysed with the social relations model (SRM). When applying the SRM to their round‐robin data, researchers are interested, for example, in estimates of the model's variance and covariance terms and/or in the effect of person‐level or dyad‐level variables on the SRM effects (Kenny, 1994).

Very often researchers have assessed multiple round‐robin variables that can refer to the same psychological construct. For example, participants were not only asked to judge the nervousness but also the calmness and relaxation of the other group members. In this case, some researchers may compute a composite of the round‐robin variables because, for example, they do not want to compute an SRM for each single round‐robin variable or they want to avoid more complex models, such as a multivariate SRM (Jorgensen et al., 2024; Kenny, 1994; Nestler, 2018), because they find the univariate model's parameters sufficient. In the standard setting, that is, when no round‐robin data are analysed, researchers would report an estimate of the reliability of the composite of the items, in addition to the results of the actual statistical model, because this allows the reader to judge whether the observed effects are distorted by measurement errors.

However, in the SRM context reporting the reliability of a round‐robin variable composite is not possible, because a scale‐type reliability index has not yet been proposed in the SRM literature. This is interesting given that in all other areas of psychology, these types of reliability indices are almost always reported. The objective of this article is to close this gap by introducing a general approach to estimate the reliability of round‐robin variable composites. To this end, we will use the recently suggested social relations structural equation model (SR‐SEM; see, Nestler et al., 2020; Nestler, Lüdtke, & Robitzsch, 2022a) to derive estimates for consistency coefficients of round‐robin variable composites. The SR‐SEM is a combination of the SRM with the SEM and hence a confirmatory factor analysis (CFA). Consequently, the SR‐CFA can be used to test hierarchically nested hypotheses about the factor loadings and measurement errors (i.e. using $\chi^{2}$ difference or likelihood ratio tests), which in turn allows to compute consistency coefficients for the most tenable factor model (e.g. coefficients for congeneric but also parallel round‐robin variables).

In what follows, we first describe the univariate SRM. We then introduce the combination of the SRM with the CFA and briefly describe how the parameters of this model can be estimated with maximum likelihood. Thereafter, we derive formulas to compute the reliability of two types of round‐robin variable composites and then illustrate the proposed methods with an empirical example. In the section that follows, we report the results of a small simulation study that investigated the suitability of the proposed SR‐CFA approach to estimate the two reliability indices depending on the number of round‐robin groups and the number of round‐robin group members. Finally, in the general discussion we outline some questions for future research and we also discuss how one of the suggested composites (i.e. person‐level composites) is related to the SRM effect estimates suggested in the SRM literature so far.

## 2 THE SOCIAL RELATIONS MODEL

The SRM is a mathematical model that is used to analyse data measured in the round‐robin design, where every member of a group is asked to rate all other group members with respect to a psychological variable. In what follows, we focus on the SRM without roles, that is, we assume that all group members are interchangeable raters (Stas et al., 2015) For example, participants may be asked to indicate how nervous each of the other group members was in a prior group discussion. Example data for a single round‐robin variable can be found in Table 1, where we present the data from n = 4 persons nested in D = 6 dyads and where we assumed, as we will do throughout the article, that there are no missing values. Of note, round‐robin data are typically assessed in a multiple round‐robin group design, that is, multiple (independent) small groups of 4‐6 persons are asked to rate each other. However, it is often assumed that there are no between‐group differences in the SRM parameters (except the mean), we, therefore, focus on a single group here. Thus, given these assumptions, there are D=.5·n·(n−1) dyads in a dataset that–in the case of a single round‐robin variable–provided n·(n−1) judgments.

**TABLE 1:** Example data of a single round‑robin group.

Perceiver	Target			
	1	2	3	4
1	–	3	4	2
2	2	–	2	3
3	3	3	–	1
4	1	4	3	–

According to the (univariate) SRM (Kenny, 1994; Kenny et al., 2006), person i's judgment of person j can be written as

(1) $$y_{ij}=\beta +p_{i}+t_{j}+r_{ij}$$

Here, β is the overall mean across all judgments (e.g. the mean of all nervousness judgments). $p_{i}$ is the perceiver effect of i that describes how much i tends to perceive others in a certain way, for example, whether i generally thinks that others are nervous. $t_{j}$ is the target effect, representing the extent j is perceived by others in a certain way. In our example, $t_{j}$ reflects how much j is judged to be nervous in general. Finally, $r_{ij}$ is the relationship effect that is a measure of is unique perception of j, for example, i's unique nervousness perception of j. In the univariate case, this component cannot be differentiated from measurement error.

Another way to introduce the SRM is to write the judgments of a dyad d in vector form. In the single round‐robin variable case, the resulting bivariate outcome vector is given by:

(2) $$\begin{array}{ll} y_{d}=\left(\begin{array}{c} y_{ij}\\ y_{ji} \end{array}\right) & =\left(\begin{array}{c} 1\\ 1 \end{array}\right)\beta +\left(\begin{array}{cc} 1 & 0\\ 0 & 1 \end{array}\right)\left(\begin{array}{c} p_{i}\\ t_{i} \end{array}\right)+\left(\begin{array}{cc} 0 & 1\\ 1 & 0 \end{array}\right)\left(\begin{array}{c} p_{j}\\ t_{j} \end{array}\right)+\left(\begin{array}{cc} 1 & 0\\ 0 & 1 \end{array}\right)\left(\begin{array}{c} r_{ij}\\ r_{ji} \end{array}\right)\\ & =X_{d}\beta +Z_{di}u_{i}+Z_{dj}u_{j}+W_{d}r_{d}\; \end{array}$$

where $y_{ij}$ is the aforementioned judgment of i concerning j, and $y_{ji}$ is j's judgment of i. Furthermore, $X_{d}$, $Z_{di}$, $Z_{dj}$ and $W_{d}$ are design matrices that relate the entries in the respective vectors to the relevant entries in the outcome vector $y_{d}$. As can be seen, the round‐robin judgments contain information about the perceiver effect and the target effect of person i as well as person j and the opposing relationship effects of a specific dyad.

Because of this, the SRM can be used to estimate the variances of the person‐level and dyad‐level effects as well as the respective covariance terms. Specifically, a multivariate normal distribution is assumed for the perceiver‐and the target effects for each person i, $u_{i}∼MVN(0,\Sigma_{u})$. In the univariate case, $\Sigma_{u}$ is

(3) $$\Sigma_{u}=\left(\begin{array}{cc} \sigma_{p}^{2} & \sigma_{pt}\\ & \sigma_{t}^{2} \end{array}\right)$$

where $\sigma_{p}^{2}$ denotes the variance of the perceiver effects. In our example, the perceiver variance measures how much perceivers differ in their average nervousness judgments. $\sigma_{t}^{2}$ is the variance of the target effects reflecting, for example, whether some targets are judged to be more nervous on average than other targets. Finally, $\sigma_{pt}$ is the covariance between the two effects. In terms of our example, a positive covariance would indicate that individuals who perceive others to be more nervous on average are also perceived to be more nervous on average.

The relationship effects are also assumed to follow a centred multivariate normal distribution, that is, $r_{d}∼MVN(0,\Sigma_{r})$. In the univariate case, $\Sigma_{r}$ is

(4) $$\Sigma_{r}=\left(\begin{array}{cc} \sigma_{r}^{2} & \sigma_{rr}\\ & \sigma_{r}^{2} \end{array}\right)$$

where the relationship variance, $\sigma_{r}^{2}$, measures the extent to which dyads differ in their unique perceptions. In our example, the relationship variance describes whether dyads differ in their unique nervousness perceptions. The relationship covariance, $\sigma_{rr}$, describes the extent to which the relationship effects are reciprocated. A positive covariance in the example would indicate that i's perception of j as nervous goes along with j's perception of i as nervous.

## 3 THE SOCIAL RELATIONS CONFIRMATORY FACTOR ANALYSIS MODEL (SR‐CFA)

In the previous section we described the univariate SRM. However, researchers often ask participants to rate several round‐robin variables, referring either to the same construct or to different constructs (see Table 2 for example data). For instance, researchers may have asked the group members to judge the items nervous, calm and relaxed (e.g. indicating neuroticism) or the items nervousness and liking. Let k index a specific round‐robin variable and let K be the total number of round‐robin variables (K = 3 in the first example). When we assume that there is no missing data, the D dyads in the dataset then provided K·n·(n−1) judgments. Similar to the univariate SRM, we can then write the judgments of a dyad d as the sum of k perceiver and target effects and 2·k relationship effects:

(5) $$\begin{array}{l} y_{d}=X_{d}\beta +Z_{di}u_{i}+Z_{dj}u_{j}+W_{d}r_{d} \end{array}$$

where $X_{d}$, $Z_{di}$, $Z_{dj}$ and $W_{d}$ are again the design matrices of respective size that relate the entries in their vectors to the relevant entries in the outcome vector $y_{d}$.

**TABLE 2:** Example data of a single round‑robin group.

(a) Raw data				
Perceiver	Target			
	1	2	3	4
1	–	3, 2, 4	4, 3, 1	2, 2, 2
2	2, 1, 3	–	2, 3, 2	3, 4, 3
3	3, 2, 2	3, 3, 4	–	1, 2, 1
4	1, 1, 1	4, 5, 3	3, 5, 5	–

(b) Dyad‑level composite				
Perceiver	Target			
	1	2	3	4
1	–	9	8	6
2	6	–	7	10
3	7	10	–	4
4	3	12	13	–

(c) Perceiver‑Level Composite	
Perceiver	
1	23
2	23
3	21
4	28

Equation (5) defines the multivariate SRM (Jorgensen et al., 2024; Nestler, 2018) that, for example, can be used to compute the SRM variance and covariance parameters for each of the single round‐robin variables and the cross‐variable covariance parameters that result from relating the SRM effects of the K round‐robin variables (e.g. the perceiver effects of nervous and calm). The model is typically estimated as a saturated model, that is, all entries in the covariance matrices $\Sigma_{u}$ and $\Sigma_{r}$ are estimated, albeit with structural equality constraints imposed on some covariances in $\Sigma_{r}$. For instance, in the bivariate case (i.e. K=2), $\Sigma_{u}$ would be a 4×4 matrix

(6) $$\Sigma_{u}=\left(\begin{array}{cccc} \sigma_{p_{1}}^{2} & \sigma_{p_{1}t_{1}} & \sigma_{p_{1}p_{2}} & \sigma_{p_{1}t_{2}}\\ & \sigma_{t_{1}}^{2} & \sigma_{t_{1}p_{2}} & \sigma_{t_{1}t_{2}}\\ & & \sigma_{p_{2}}^{2} & \sigma_{p_{2}t_{2}}\\ & & & \sigma_{t_{2}}^{2} \end{array}\right)$$

where $\sigma_{p_{1}p_{2}}$, $\sigma_{p_{1}t_{2}},\sigma_{t_{1}p_{2}}$ and $\sigma_{t_{1}t_{2}}$ are the cross‐variable‐covariance parameters, and all other entries refer to the SRM variance and covariance parameters for each of the two single round‐robin variables.

While the multivariate SRM provides parameters that can be of substantive interest, a disadvantage of the model is that it cannot be used to test structural hypotheses concerning the multivariate relations between the SRM effects. For instance, when it is of interest to test whether the relationships behind the SRM effects of the round‐robin variables nervous, calm, and relaxed are due to a single latent perceiver neuroticism factor and/or due to a latent target neuroticism factor, the multivariate model is not suitable. Rather a combination of the SRM with the CFA has to be used. In this SR‐CFA (see Nestler et al., 2020; Nestler, Lüdtke, & Robitzsch, 2022a), the measurement model is defined by writing the *whole* vector of round‐robin judgments y in terms of the person effects for each individual i and the relationship effects for each dyad d:

(7) $$y=X\beta +\overset{I}{\underset{i=1}{\sum }}Z_{i}u_{i}+\overset{D}{\underset{d=1}{\sum }}W_{d}r_{d},$$

where β, $u_{i}$ and $r_{d}$ are defined as before. X, $Z_{i}$ and $W_{d}$ are again design matrices that are now defined for the whole judgment vector (in contrast to Equation 5).

The CFA part of the model is defined for the vector of person effects *and* the vector of dyad effects, respectively, by assuming that both are linear functions of latent person‐and latent dyad factors, respectively:

(8) $$\begin{array}{ll} u_{i} & =\Lambda_{u}\eta_{u_{i}}+\varepsilon_{u_{i}}\\ r_{d} & =\Lambda_{r}\eta_{r_{d}}+\varepsilon_{r_{d}}\; \end{array}$$

Here, $\Lambda_{u}$ is a $2K\times n_{u}$ matrix of factor loadings, $\eta_{u_{i}}$ is an $n_{u}\times 1$ vector of latent perceiver and target effects for person i and $\varepsilon_{u_{i}}$ is a 2K×1 vector of residual terms for i. Similarly, $\Lambda_{r}$ is a $2K\times n_{d}$ matrix of factor loadings that relate the latent relationship vectors in the $n_{d}\times 1$ vector to the 2K relationship effects of the K measures. $\varepsilon_{r_{d}}$ is a 2k×1 vector of residual terms for dyad d. Note that in contrast to standard CFA models, the factor models in Equation (8) do not contain an intercept vector because the factor model is defined for mean‐centred random variables (see Nestler et al. (2020), for a more thorough discussion of the identification of latent SRM factor means).

When we assume that $\eta_{u_{i}}$, $\varepsilon_{u_{i}}$, $\eta_{r_{d}}$ and $\varepsilon_{r_{d}}$ are multivariate normal random variables with expectation zero and covariance matrix $\Phi_{u}$, $\Psi_{u}$, $\Phi_{r}$ and $\Psi_{r}$, the covariance matrices of the person‐level and the dyad‐level effects, respectively, are (e.g., Bollen, 1989)

(9) $$\begin{array}{ll} \Sigma_{u} & =\Lambda_{u}\Phi_{u}\Lambda_{u}^{\prime }+\Psi_{u}\\ \Sigma_{r} & =\Lambda_{r}\Phi_{r}\Lambda_{r}^{\prime }+\Psi_{r}\; \end{array}$$

Furthermore, when we also assume that the person‐level effects and the dyad‐level effects are independent, as it is typically done, the covariance matrix of the round‐robin vector y is 
(10)

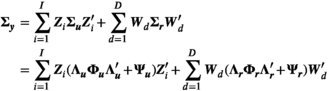

and the expectation of y is $\mu_{y}=X\beta$.

We note that the univariate and the multivariate SRM are special cases of the SR‐CFA by defining $\Lambda_{u}$ ($\Lambda_{r}$) to be an identity matrix and setting $\Psi_{u}$ ($\Psi_{r}$) to zero. Finally, the model in Equation (7) can also be written in more compact form

(11) $$\begin{array}{ll} y & =X\beta +Zu+Wr\; \end{array}$$

where $Z=(Z_{1},\text{⋯}\;,Z_{n})$, $u={\left(u_{1},\text{⋯}\;,u_{I}\right)}^{\prime }$, $W=(W_{1},\text{⋯}\;,W_{D})$ and $r={\left(r_{1},\text{⋯}\;,r_{D}\right)}^{\prime }$. This highlights the close connection between the SR‐CFA (and also the SRM) with the mixed‐effects model (Nestler, 2016, 2018; Skrondal & Rabe‐Hesketh, 2004), and it also shows the special role of the relationship effects. For example, Wr is just a vector of residual terms in the univariate SRM because W is an identity matrix. This explains why in the univariate SRM, it is not possible to differentiate between true relationship effects and measurement error.

### 3.1 Estimation

Several approaches were suggested in the literature to estimate the parameters of univariate and the multivariate SRM (see Jorgensen et al. (2024), for an overview), ranging from method‐of‐moments‐type estimators (Bond & Lashley, 1996; Lashley & Bond, 1997; Warner et al., 1979), maximum likelihood (ML) and restricted ML estimators (see e.g., Gill & Swartz, 2001; Nestler, 2016; Snijders & Kenny, 1999) to Bayesian approaches (e.g., Gill & Swartz, 2001; Hoff, 2005; Lüdtke et al., 2013, 2018). Also, researchers showed how to estimate the SRM parameters in commonly used structural equation model software (Kenny, 1998; Jendryczko & Nussbeck, 2022a, 2022b).

For the SR‐CFA, we suggested using an ML estimator (see Nestler et al., 2020) that is implemented in the R package srm(Nestler, Robitzsch, & Luedtke, 2022b) and that yielded acceptable biases and coverage rates in a simulation study, except when only a few small round‐robin groups are available for parameter estimation (e.g. 15 five‐person groups). Let θ be the vector of the SR‐CFA parameters that we want to estimate with the data. When we assume that all latent variables are normally distributed, it follows that the vector of round‐robin judgments is also normal with expectation $\mu_{y}$ and covariance matrix $\Sigma_{y}$ (see Equation 10). The log‐likelihood of the round‐robin data given the parameter vector θ is then given by

(12) $$l(\theta )\propto -\frac{1}{2}\log(|\Sigma_{y}^{-1}|)-\frac{1}{2}{\left(y-\mu_{y}\right)}^{\prime }\Sigma_{y}^{-1}(y-\mu_{y})$$

where we ignored constant terms.

Nestler et al. (2020) used Equation (12) to derive the score equation and the expected Fisher information matrix to implement a Fisher scoring algorithm. For instance, the score equation is

(13) $$\frac{\partial l}{\partial \theta_{h}}=-\frac{1}{2}\text{tr}\left(\Sigma_{y}^{-1}\frac{\partial \Sigma_{y}}{\partial \theta_{h}}\right)+\frac{1}{2}{\left(y-\mu_{y}\right)}^{\prime }\Sigma_{y}^{-1}\frac{\partial \Sigma_{y}}{\partial \theta_{h}}\Sigma_{y}^{-1}(y-\mu_{y})+{\left(y-\mu_{y}\right)}^{\prime }\Sigma_{y}^{-1}\frac{\partial \mu_{y}}{\partial \theta_{h}}$$

where finding the derivatives of $\Sigma_{y}$ can be reduced to finding the derivatives of $\Sigma_{u}$ and $\Sigma_{r}$

(14) $$\frac{\partial \Sigma_{y}}{\partial \theta_{h}}=\overset{I}{\underset{i=1}{\sum }}Z_{i}\frac{\partial \Sigma_{u}}{\partial \theta_{h}}Z_{i}^{\prime }+\overset{D}{\underset{d=1}{\sum }}W_{d}\frac{\partial \Sigma_{r}}{\partial \theta_{h}}W_{d}^{\prime }$$

Formulas to compute the derivatives of $\Sigma_{u}$ and $\Sigma_{r}$ are available in the SEM literature (see e.g., Bollen, 1989; Mulaik, 2009).

## 4 RELIABILITY OF COMPOSITES OF ROUND‐ROBIN VARIABLES

We now show how the SR‐CFA can be used to estimate the reliability of a composite of round‐robin variables. In our exposition, we rely on the classical definition of reliability, that is, the proportion of the variance of the true scores to the variance of the observed scores (McDonald, 1999; Raykov & Marcoulides, 2011), $\rho_{Y}=\sigma_{T}^{2}/\sigma_{Y}^{2}$. Furthermore, we assume that person i has rated person j on K round‐robin variables, for example, i has rated j on the three items nervous, calm and relaxed (see Table 2a for some example data for a single round‐robin group). We focus on two possible types of composites: First, researchers may calculate the sum of the K round‐robin judgments of person i concerning person j. For example, they may sum i's three ratings of j on the variables nervous, calm and relaxed (see Table 2b). Since the composite refers to the judgments of i about j, that is, the combination ij, we call this the dyad‐level composite in what follows. Second, researchers may compute the sum of all judgments of a perceiver (or a target) across all targets (or perceivers). In our example, they may calculate the perceiver‐level neuroticism composite by calculating the sum of all ratings of i across all targets and items (see Table 2c for an example). Since this composite refers to the person, we call it the person‐level composite in the following expositions.

We believe there are several situations in which researchers may use such composites. With regard to the dyad‐level composite, to begin with, researchers may simply want to compute a univariate SRM because they are not interested in the multivariate relationships between the SRM effects, because they found the univariate SRM easier to interpret since it has fewer parameters or because they want to use the dyadic round‐robin variable in another statistical model that cannot be easily combined with the SRM (e.g. the dyadic neuroticism composite in a social network model investigating friendship development, see Jorgensen et al. (2018) and van Zalk et al. (2020)). Similarly, person‐level composites may be computed because researchers may want to use them in later person‐level analyses. One could argue that a better approach would be to do such analyses within the SR‐SEM. However, this might not always be possible because the full model may be too large or complex to be estimated in the available sample (e.g. in addition to the SR‐CFA the model may also contain several latent person‐level variables). Alternatively, one could argue that one could also fit a SR‐CFA in this case and then use the respective factor scores of this model in the analyses. However, similar to composites, factor scores are also *estimates* for which—strictly speaking—a reliability estimate should be provided. Whatever the reason for computing a composite, the reliability of this composite might then be reported because it indicates how strongly the respective composite is prone to measurement error. This information allows readers to judge whether and to what extent observed effects may be distorted by measurement error.

### 4.1 Dyad‐level composite

To simplify the following exposition, we assume that the round‐robin variables are mean‐centred variables (e.g. β = 0). Furthermore, we collect the K judgments of i concerning j in a vector and assume, in accordance with classical test theory, that $y_{ij}$ can be written as

(15) $$y_{ij}=\tau_{ij}+\epsilon_{ij}$$

where $\tau_{ij}$ is a K×1 vector of true scores reflecting the measurement error free part of $y_{ij}$ and $\epsilon_{ij}$ is a vector of measurement errors. In addition, the true scores and measurement errors are assumed to be uncorrelated random variables.

Furthermore, we assume that the true scores are the sum of true perceiver‐and target effects and true relationship effects (see e.g. Raykov & Zhang, 2024):

(16) $$\tau_{ij}=\Lambda_{p}p_{i}+\Lambda_{t}t_{j}+\Lambda_{r}r_{ij}$$

Here, $p_{i}$ is an $n_{p}\times 1$ vector of true perceiver effects, $t_{j}$ is an $n_{t}\times 1$ vector of true target effects, and $r_{ij}$ is an $n_{r}\times 1$ vector of true relationship effects. $\Lambda_{p}$, $\Lambda_{t}$, and $\Lambda_{r}$ are factor loading matrices of respective size that each determine how strong the respective person‐ or dyad‐level effect is represented in the overall true score. Finally, we also assume that the measurement errors can be decomposed into a component due to the perceiver, the target and the dyad:

(17) $$\epsilon_{ij}=\epsilon_{i}^{p}+\epsilon_{j}^{t}+\epsilon_{ij}^{r}$$

where the superscript indicates the respective component.^1^

Substituting Equations (16) and (17) into Equation (15) yields:

(18) $$y_{ij}=\Lambda_{p}p_{i}+\Lambda_{t}t_{j}+\Lambda_{r}r_{ij}+\epsilon_{i}^{p}+\epsilon_{i}^{t}+\epsilon_{ij}^{r}$$

and—using the SR‐CFA (see Equations 9 and 10)—the covariance matrix of $y_{ij}$ is an additive decomposition of the covariance matrix of the true scores and the covariance matrix of the errors, which in turn are the sum of the single effects' matrices: 
(19)

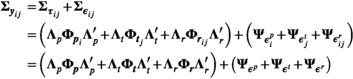

because the perceiver and target effect components are unrelated within a dyad ij, because they are not associated with the relationship effects, and because all SRM effects are unrelated with the measurement errors. Furthermore, the third line follows from assuming that the covariance matrix of the perceiver effects (target effects) is the same across all n perceivers (targets), that the covariance matrix of the relationship effects is the same across all D dyads and that the covariance matrices of the measurements errors are also the same across all persons and all dyads, respectively. Note that $\Phi_{p}$ ($\Phi_{t}$) contains the variance terms of the latent perceiver (target) effects and their covariances; they do *not* contain the perceiver‐target covariance terms (e.g. $\sigma_{p_{1}t_{1}}$ in Equation 6), because we consider the K judgments of a single dyad ij. The same applies to the matrix of the relationship effects, that is, the matrix only contains the covariance terms across indicators but within the same dyad ij. However, this does not imply that these covariances are set to zero or can be ignored when fitting the SR‐CFA. In fact, the full model should be fitted to the data to prevent a (potentially) biased estimate of a reliability coefficient. Finally, $\Psi_{\epsilon^{p}}$, $\Psi_{\epsilon^{t}}$ and $\Psi_{\epsilon^{r}}$ also do not contain these perceiver‐target or across‐dyad covariance terms. However, these matrices may contain covariance terms that reflect correlated residuals for the respective SRM effects (e.g. two residual perceiver effects are assumed to be correlated).

Notably, the model suggested in Equations ((15), (16), (17), (18), (19)) is very general, because we do not assume that the factor model of the perceiver effects, the factor model of the target effects, and the model of the relationship effects is unidimensional (i.e. $n_{p}=n_{t}=n_{r}=1$; see Bögemann et al. (2025), Rau et al. (2021), Srivastava et al. (2010) for applications with multifactorial SRM effect models). Furthermore, we also do not assume that the number of latent factors is the same across the three effects of the SRM (i.e. $n_{p}=n_{t}$) and we allow for correlated residuals. However, for this generality to apply the factor models have to be identified and the estimates of the model parameters must be meaningful (no Heywood cases, e.g. residual variance terms less than zero). Depending on the number of measured round‐robin variables or model convergence it may therefore be the case that only single‐factor models can be estimated. However, we use this complex model to derive a formula for determining reliability that is as general as possible.

Specifically, we can use Equations (15) and (19) to derive the reliability of the dyad‐level composite, because the sum of the round‐robin variables for dyad ij can be written as

(20) $$\begin{array}{l} y_{ij1}+\text{⋯}+y_{ijK}=1^{\prime }y_{ij}=1^{\prime }\tau_{ij}+1^{\prime }\epsilon_{ij} \end{array}$$

A general expression for the reliability of the sum score then is (Raykov & Shrout, 2002; Raykov & Zhang, 2024)^2^: 
(21)

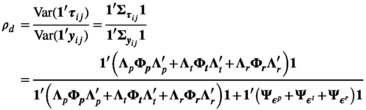

To gain insight into this formula, we consider some special cases. When we assume, to begin with, a one‐factor model for each SRM effect, that the factor loadings can differ across the K round‐robin variables (e.g. a congeneric unidimensional scale, see McDonald, 1999; Raykov & Marcoulides, 2011; Raykov & du Toit, 2005) and that there are no residual correlations, Equation (20) simplifies to 
(22)

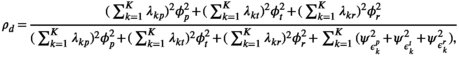

where we assume that the first loading is set to 1 for each SRM effect to identify each factor model. Furthermore, when we additionally assume that the factor loadings and the error variances are invariant across the SRM effects and that both are the same for each variable (i.e. parallel items, see McDonald, 1999), Equation (21) is

(23) $$\rho_{d}=\frac{{\left(\sum_{k=1}^{K}\lambda_{k}\right)}^{2}(\phi_{p}^{2}+\phi_{t}^{2}+\phi_{r}^{2})}{{\left(\sum_{k=1}^{K}\lambda_{k}\right)}^{2}(\phi_{p}^{2}+\phi_{t}^{2}+\phi_{r}^{2})+3\cdot K\cdot \psi_{\epsilon }^{2}},$$

indicating that the reliability of the composite is larger, the more round‐robin variables have been assessed.

### 4.2 Person‐level composites

Let us now turn to the situation where researchers want to sum all the judgments of a perceiver i concerning *all* targets; the situation in which one is interested in the sum of all judgments across all perceivers can be treated in the same way. When there are no missing values, this is a sum of K·(n−1) judgments. To derive the formula for calculating the reliability, we again collect the K judgments of i concerning j in a vector and write this vector as a sum of true score vector and a vector containing measurement errors:

(24) $$y_{i}=\tau_{i}+\epsilon_{i}$$

The difference between Equations (15) and (24) is that in the former, the vector $y_{ij}$ contains the K judgments of a i concerning j, while vector $y_{i}$ contains all judgments of perceiver i (i.e. $y_{i}=(y_{i11},\text{…},y_{i1K},y_{i21},\text{…},y_{in1},\text{…},y_{inK})$).

The model for the vector of true scores and the errors, respectively, is

(25) $$\begin{array}{ll} \tau_{i} & =Z_{p}p_{i}+Z_{t}t_{j}+Z_{r}r_{ij}=(1_{K}⊗\Lambda_{p})p_{i}+(I_{K}⊗\Lambda_{t})t_{j}+(I_{K}⊗\Lambda_{r})r_{ij}\\ \epsilon_{i} & =Z_{\epsilon ,p}\epsilon_{i}^{p}+Z_{\epsilon ,t}\epsilon_{j}^{t}+Z_{\epsilon ,r}\epsilon_{ij}^{r}=(1_{K}⊗I_{K})\epsilon_{i}^{p}+(I_{K}⊗I_{K})\epsilon_{j}^{t}+(I_{K}⊗I_{K})\epsilon_{ij}^{r}\; \end{array}$$

where ⊗ is the Kronecker product and $I_{K}$ is a K×K identity matrix. We have to represent the model in a more complex way because we have to ensure that the entries in the latent variable vectors are linked to the correct entries in $\tau_{i}$ and $\epsilon_{i}$, respectively.

Having defined the true score model, we can now proceed in the same way as in the case of the reliability of the dyad‐level composite. The resulting reliability coefficient then is:

(26) $$\begin{array}{ll} \rho_{p} & =\frac{1^{\prime }Z_{p}\Phi_{p}Z_{p}^{\prime }1}{1^{\prime }\left(Z_{p}\Phi_{p}Z_{p}^{\prime }+Z_{t}\Phi_{t}Z_{t}^{\prime }+Z_{r}\Phi_{r}Z_{r}^{\prime }\right)1+1^{\prime }\Sigma_{\epsilon }1}\; \end{array}$$

where 1 is a K·(n−1)×1 vector containing ones and $\Sigma_{\epsilon }$ is the sum of the unique covariance matrices (i.e. $Z_{\epsilon ,p}\Psi_{p}Z_{\epsilon ,p}^{\prime }+\text{…}$). In particular, the additive decomposition results again because $y_{i}$ does not contain the judgments of the opposing dyads (i.e. $y_{jik}$). From this it also follows that $\Sigma_{p}$ ($\Sigma_{t}$) contains the variance terms of the latent perceiver (target) effects and their covariances only; they do *not* contain the perceiver‐target covariance terms. The same applies to the matrix of relationship effects.

To provide further insights into the computation of $\rho_{p}$, we consider the special case that only a single round‐robin variable has been assessed and researchers consider the sum of the judgments of perceiver i regarding all targets j. In this case, Equation (26) reduces to

(27) $$\begin{array}{ll} \rho_{p} & =\frac{\phi_{p}^{2}}{\phi_{p}^{2}+\phi_{t}^{2}/(n-1)+\phi_{rr}^{2}/(n-1)}\; \end{array}$$

because K=1 implies a one‐factor model for each SRM‐effect where the factor loading of the (single) indicator is 1 and that $\Sigma_{\epsilon }=\Phi_{r}$ (i.e. in the case of a single variable, one cannot differentiate between true relationship and measurement error variance).

Finally, similar formulas can be obtained for the sum of all the judgments of a target i by all perceivers. The resulting reliability is

(28) $$\begin{array}{ll} \rho_{t} & =\frac{1^{\prime }Z_{t}\Phi_{t}Z_{t}^{\prime }1}{1^{\prime }\left(Z_{p}\Phi_{p}Z_{p}^{\prime }+Z_{t}\Phi_{t}Z_{t}^{\prime }+Z_{r}\Phi_{r}Z_{r}^{\prime }\right)1+1^{\prime }\Sigma_{\epsilon }1}\; \end{array}$$

which reduces to

(29) $$\begin{array}{ll} \rho_{t} & =\frac{\phi_{t}^{2}}{\phi_{t}^{2}+\phi_{p}^{2}/(n-1)+\phi_{rr}^{2}/(n-1)}\; \end{array}$$

in the single variable case.

### 4.3 Estimation of the reliability indices

To obtain an estimate of a specific reliability index in empirical applications, one has to obtain estimates of the model matrices contained in Equations (21), (26) and/or (28). To this end, the SR‐CFA can be fit as specified in Equation 10 and can use the relevant elements of $\Lambda_{u}$, $\Phi_{u}$, $\Psi_{u}$, $\Lambda_{r}$, $\Phi_{r}$ and $\Psi_{r}$ to calculate the reliability index in which one is interested.

Importantly, we presented formulas for the most general case but also for a number of special cases that resulted from introducing a number of restrictions on the model parameters (e.g. equal factor loadings across measures or equality constraints across levels). It is possible to estimate the SR‐CFA with these restrictions and to compare the fit of the restricted model with the fit of a less restricted model using $\chi^{2}$ difference or likelihood ratio tests similar to what is known from the standard CFA (Bollen, 1989; Kline, 2016). Alternatively, the AIC can be used to compare the fit of both nested and non‐nested models. Whatever approach is taken, the SR‐CFA allows the most tenable model to be found for the available data, although the quality of the decision will, among other things, depend on the size of the sample (i.e. the number of round‐robin group members) and on whether the distributional assumptions are met.

Furthermore, when we introduced the formulas for the covariance matrix of a composite, we mentioned that these matrices do not contain the covariances between the perceiver and target effects for each measure K, the covariances between the opposing relationship effects for each measure K and the covariances between the error terms of opposing dyads for each measure K, for example, cov$\left(\epsilon_{ijk},\epsilon_{jik}\right)$, because we focus on a single dyad ij. However, this does not imply that these covariances can be ignored when fitting the SR‐CFA. In fact, the full model should be fitted to the data to prevent a (potentially) biased estimate of a reliability coefficient.

Finally, we described above how to obtain a reliability coefficient with the estimated SR‐CFA parameters, but the approach does not provide information on the certainty of the estimate. To obtain a standard error estimate and a large sample 100(1−α)% confidence interval, we suggest applying the multivariate delta theorem Casella and Berger (2002), because each reliability coefficient is a function of the estimates of the SR‐CFA parameters that are, at least asymptotically, multivariate normal distributed. Specifically, we can express each of the introduced reliability coefficient as a function of the model parameters

(30) $$\begin{array}{ll} \rho (\theta ) & =\frac{g(\theta )}{h(\theta )}\; \end{array}$$

In the case of $\rho_{p}$ (see Equation 26) the function in the numerator and the denominator, respectively, is:

(31) $$\begin{array}{ll} g(\theta ) & =1^{\prime }Z_{p}\Phi_{p}Z_{p}^{\prime }1\\ h(\theta ) & =1^{\prime }\left(Z_{p}\Phi_{p}Z_{p}^{\prime }+Z_{t}\Phi_{t}Z_{t}+Z_{r}\Phi_{r}Z_{r}^{\prime }\right)1+1^{\prime }\Sigma_{\epsilon }1\; \end{array}$$

The variance of a coefficient can then be obtained with:

(32) $$\begin{array}{ll} \mathrm{Var}(\rho ) & =\frac{\partial \rho (\theta )}{\partial \theta }\text{Cov}(\theta )\frac{\partial \rho {\left(\theta \right)}^{\prime }}{\partial \theta }\; \end{array}$$

where Cov(θ) is the asymptotic covariance matrix of the model parameters, $\frac{\partial \rho (\theta )}{\partial \theta }$ is the derivative of ρ(θ) with respect to the model parameters. This derivative can be obtained with (e.g., Casella & Berger, 2002; Raykov & du Toit, 2005)

(33) $$\begin{array}{l} \frac{\partial \rho (\theta )}{\partial \theta }=\frac{1}{h(\theta )}\left(\frac{\partial g(\theta )}{\partial \theta }-\rho (\theta )\frac{\partial h(\theta )}{\partial \theta }\right) \end{array}$$

where the derivative of the denominator is the gradient of the SR‐CFA. Both gradients can be obtained analytically, or one can use finite‐difference methods to calculate them. Finally, to obtain a reliability coefficient and its standard error, the estimated model parameters are inserted into Equations (30), (32) and (33).

## 5 EXAMPLE

To illustrate our approach, we use part of the data from the EXACT study (see Nestler et al. (2020), who also used this data). The EXACT study was a zero‐acquaintance round‐robin experiment consisting of two group sessions. In each session, the group members of 24 round‐robin groups (consisting of five to six individuals each, with an overall n = 141) were asked to introduce themselves briefly to their round‐robin group members. Afterwards, they were asked to judge the other members of the group on 10 adjectives that encompass different dimensions of interpersonal personality. All judgments were assessed on 9‐point Likert‐type scales (ranging from 1 = not at all to 9 = very much). For this illustration, we used three of these judgments (shy, nervous, calm) and calculated an estimate of the reliability of the dyad‐level composite (i.e. the sum of the three items for each dyad ij), the composite for a perceiver (i.e. the sum of all perceiver judgments across all three items) and the composite of the target.

To estimate the three reliability coefficients, we first fitted the SR‐CFA in which a one‐factor model was defined for the three round‐robin judgments shy, nervous and calm. Specifically, we assumed that the perceiver effects contained in the three judgments would load on one latent perceiver‐effect factor reflecting neuroticism. The same one‐factor model was also posited for the target effects. Additionally, we assumed that the two latent factors were correlated, and we allowed the perceiver‐effect and target‐effect residual terms of a certain round‐robin variable to be correlated. Finally, a one‐factor model was also defined for the relationship effects. We used the R package srm to obtain the ML parameter estimates. All R codes and data can be downloaded from the Open Science Framework (OSF, https://osf.io/tde49/).

The resulting coefficients, together with their standard errors, are shown in Table 3. With these parameter estimates, we computed the three reliability estimates together with their standard error. The resulting values, including a 95% per cent confidence interval can be found in Table 4. The dyad‐level composite shows a fairly high reliability, although only three items were assessed. The coefficients for the person‐level composites are smaller, whereby the reliability of the perceiver‐level composite is smaller than the composite of the targets. However, the perceiver effect factor is also the factor with the smallest variance (see Table 3) which may explain the difference between the two coefficients.

**TABLE 3:** Results of the SR‐CFA for the illustrative data.

Parameter	Person‐Level		Parameter	Dyad‐Level	
	Est.	SE		Est.	SE
$\lambda_{2p}$	1.89	.48	$\lambda_{2r}$	1.35	.12
$\lambda_{3p}$	1.21	.29	$\lambda_{3r}$	1.13	.09
$\lambda_{2t}$	.85	.09			
$\lambda_{3t}$	.70	.08			
$\phi_{p}^{2}$	.18	.09	$\phi_{r}^{2}$	.95	.14
$\phi_{t}^{2}$	1.23	.27			
$\phi_{pt}$	−.14	.08	$\phi_{rr}$	.00	.08

*Note*: Unstandardized parameter estimates are shown in the table. The variance and covariance parameters of the person‐level and dyad‐level residual error terms are not shown in the table. Abbreviation: SE, standard error.

**TABLE 4:** Reliability estimates for three composites for the illustrative data.

Composite	Estimate	SE	95% CI
Dyad	.85	.01	[0.83, 0.88]
Perceiver	.38	.07	[0.25, 0.51]
Target	.67	.04	[0.58, 0.77]

Abbreviations: CI, confidence interval; SE, standard error.

In most psychological research, reliability coefficients around .70 are considered indicative of sufficient reliability (McDonald, 1999; Raykov & Marcoulides, 2011). However, because reliability for multi‐item composites has never been reported in SRM research–due to the lack of appropriate indices–it is difficult to evaluate whether the obtained values are sufficient. But even without established benchmarks, we think that the reliabilities provide valuable comparative information. For example, if the person‐level composites were used as predictors in subsequent analyses, we would expect effects involving perceiver‐level neuroticism to be more attenuated by measurement error than effects involving target‐level neuroticism.

## 6 SIMULATION STUDY

We conducted a small simulation study to examine the performance of our SR‐CFA reliability estimation approach. We simulated data for 15 or 50 round‐robin groups with either 5 or 10 group members. The population model was a SR‐CFA model in which one latent perceiver‐effect factor, one latent target‐effect factor, and one latent relationship‐effect factor determined the responses to *three* round‐robin variables. The model's parameters were set to the same values as in Nestler et al. (2020). That is, the model matrices for the person‐level effects were

$$\Lambda_{u}^{T}=\left(\begin{array}{cccccc} 1.0 & 1.2 & .7 & .0 & .0 & .0\\ .0 & .0 & .0 & 1.0 & .6 & .6 \end{array}\right)\text{,}\;\Phi_{u}=\left(\begin{array}{cc} .40 & .05\\ .05 & .20 \end{array}\right)$$

and

$$\Psi_{u}=\left(\begin{array}{cccccc} .20 & .00 & .00 & .05 & .00 & .00\\ .00 & .20 & .00 & .00 & .00 & .00\\ .00 & .00 & .20 & .00 & .00 & .03\\ .05 & .00 & .00 & .10 & .00 & .00\\ .00 & .00 & .00 & .00 & .10 & .00\\ .00 & .00 & .03 & .00 & .00 & .10 \end{array}\right),$$

and for the dyad‐level effects the model matrices were

(34) $$\Lambda_{r}^{T}=\left(\begin{array}{cccccc} 1.0 & .0 & .8 & .0 & 1.4 & .0\\ .0 & 1.0 & .0 & .8 & .0 & 1.4 \end{array}\right)\text{,}\;\Phi_{r}=\left(\begin{array}{cc} .60 & .15\\ .15 & .60 \end{array}\right)$$

and

$$\Psi_{r}=\left(\begin{array}{cccccc} .30 & .00 & .00 & .00 & .00 & .00\\ .00 & .30 & .00 & .00 & .00 & .00\\ .00 & .00 & .50 & .10 & .00 & .00\\ .00 & .00 & .10 & .50 & .00 & .00\\ .00 & .00 & .00 & .00 & .40 & -.20\\ .00 & .00 & .00 & .00 & -.20 & .40 \end{array}\right).$$

The population parameters, together with the number of round‐robin group members determine the true reliability coefficients for the person‐level composites. For the perceiver‐level composite, the true reliability was about .55 and .68 when the number of group members was 5 and 10, respectively. For the target‐level composites the true values were .25 and .39. For the dyad‐level composite, finally, the true reliability was about .84 in all four simulation conditions.

In each simulation condition, we generated 2,000 samples. The srm package was used to obtain the model parameter estimates in each replication. These parameters were then used to obtain the three reliability estimates, and the standard error of a reliability coefficient was calculated using the multivariate delta theorem (see above). As performance measures, we used the median of a reliability estimate as a robust variant of the mean, and the median absolute deviation (MAD), as implemented in the R function mad(), which measures the spread of estimates around their median, as a robust variant of the standard deviation. These robust measures were chosen to reduce the influence of outlying (i.e. extreme) reliability estimates within a simulation condition. We also examined the observed coverage of the 95% confidence intervals. These were obtained by computing the confidence interval with the standard error of the respective reliability estimate in each replication. The coverage was then coded 1 if the true parameter value (i.e. the respective true reliability coefficient) was included and 0 otherwise.

Table 5 presents the results of the simulation. As can be seen, the median estimate was close to the true value for all three reliability coefficients in all simulation conditions. The exception was the reliability of the target‐level composite that substantially differed from the true value in the condition with 5 round‐robin group members and fifteen round‐robin groups (i.e. the median relative bias was about 13%). In this condition, all three estimates are more variable than in the other three conditions as indicated by the MAD results. Furthermore, there was also substantial undercoverage for all three reliability estimates in this condition. However, with a larger number of groups and/or a larger number of round‐robin group members, the estimates became more stable and the CRs were close to nominal.

**TABLE 5:** Median estimate (Md), median absolute deviation (MAD) and coverage rate (CR) of the three SRM reliability estimates depending on the number of round‐robin groups and the number of persons within each round‐robin group.

*n*	G	Perceiver				Target				Dyad			
		True	Md	MAD	CR	True	Md	MAD	CR	True	Md	MAD	CR
5	15	.55	.561	.089	88.4	.25	.284	.144	81.6	.84	.848	.032	89.3
	50	.55	.553	.046	94.0	.25	.250	.062	93.8	.84	.838	.013	93.6
10	15	.68	.684	.039	94.8	.39	.390	.063	94.9	.84	.836	.012	95.2
	50	.68	.684	.021	95.2	.39	.386	.035	95.4	.84	.834	.006	95.3

Abbreviations: *G*, Number of round‐robin groups; *N*, Number of round‐robin group members.

## 7 DISCUSSION

The goal of the present article was to derive reliability indices for composites of round‐robin variables because applied researchers find such composites useful and very often report them in the single‐level and the multilevel‐setting. Based on methodological research that places reliability into the CFA framework, we derived very general formulas for computing a scale‐type reliability for two round‐robin composites in which—amongst other things—the number of factors can differ between the SRM effects and/or in which the factor loadings can differ between the two types of person‐level SRM effects. We also showed how the reliability of the different composites including their standard errors can be calculated, illustrated our explanations with an example, and we also reported the results of a simulation study establishing the suitability of the suggested estimation approach in realistic data situations.

### 7.1 Questions for future research

The generality of our approach may raise questions about how to interpret a composite from a substantive point of view, for example, when there are multiple latent factors for a person‐level effect or when differential factor loadings are allowed across the perceiver and target effects. Whether and, if so, how these complexities can be resolved is certainly an interesting question for future research from both a substantive and a methodological point of view. This also applies to the debate about whether equality constraints on the loadings of perceiver and target effect factors should be imposed to enable meaningful comparisons (see Jendryczko & Nussbeck (2025), for such a proposition). However, regardless of how these issues will be solved, the approach presented here for determining the reliability indices is very general; it can thus be applied when factor loadings are constrained to be equal or not.

Furthermore, by building on this general framework, several other interesting questions may warrant further investigation. For all three composites, to begin with, we presented formulas from which simpler variants could be derived when the parameters of the SR‐CFA model are restricted in a specific way. In the unidimensional case, for example, this resulted in omega‐type reliability measures (McDonald, 1999; Raykov & Marcoulides, 2011), which can be transferred to alpha‐type reliability measures by further restrictions. In the psychometric literature, it is often argued that an omega‐type measure should be preferred to an alpha‐type measure (see McNeish (2018), but also Raykov and Marcoulides (2019)), although the empirical differences between the two coefficients in the one‐dimensional case are rather small (Savalei & Reise, 2019). However, these considerations have not been applied to the SRM, possibly because no reliability measure has been proposed for composites to date. Future research could investigate how important individual model assumptions actually are when obtaining the different types of reliabilities. For example, it could be examined whether the assumption of tau equivalence is important as the assumption of one‐dimensionality and measurement invariance across persons and dyads.

The approach considered here yields model‐based reliability coefficients; therefore, it is crucial to select the correct underlying latent variable model, and the SR‐CFA can be used for these model selection decisions. However, so far no methodological research has examined how well model selection works in different SRM data contexts. Furthermore, it is not clear whether, and if so how much, the selection decisions are influenced by smaller and larger misspecifications in the other‐level model (e.g. correct person‐levels, but an incorrect dyad‐level model). Thus, we believe that future research should investigate these important questions, which are also relevant for other applications of the SRM independent of the reliability question.

The ideas presented here may also be extended at the model level. For example, Raykov and Zhang (2024) use the CFA to investigate how items should be weighted in a composite such that this composite has maximal reliability. In our view, it might be interesting to transfer these considerations to the SRM context. In addition, there are a variety of approaches to account for measurement error in statistical analysis that uses a composite or a single variable (see Buonaccorsi (2010) or Lüdtke et al. (2018)). Although it is not necessary from the perspective of the SR‐SEM to make such error corrections, because the influences of and the effects on SRM latent variables can be modelled directly in the SR‐SEM, we have described several situations in which simpler statistical models are preferred. Hence, an interesting question for future research might be to investigate how to best implement these error correction methods in the case of round‐robin data.

The present article adopts a factor‐analytic perspective on determining reliability coefficients for the SRM. An alternative conceptual framework for assessing the reliability of composite variables is generalizability theory (Brennan, 2001; Cronbach et al., 1972). Within this framework, items and ratings are treated as exchangeable under a domain sampling model (Tryon, 1957). In the specific case of a single round‐robin variable, the reliability of the sum of all judgments of a target (see Equation 29) can be interpreted as an inter‐rater reliability, whereas the reliability of the sum of the judgments of a perceiver (see Equation 27) represents an intra‐rater reliability. These reliability coefficients quantify the generalizability across ratings obtained for a person (inter‐rater) or produced by a person (intra‐rater). Furthermore, the corresponding multi‐item composites (see Equations 26 and 28) indicate the generalizability of scores across items and ratings (see Ten Hove et al. (2025) for a discussion). Previous methodological research has pointed out that generalizability coefficients can also be obtained using a SEM approach (Marcoulides, 1996; Vispoel et al., 2023). However, it should be emphasized that, in the case of ML estimation in SEM, this approach assumes that a unidimensional model with parallel items holds in the population (i.e. has perfect fit). When the model is misspecified, unweighted least squares (ULS) should be used to determine the corresponding generalizability coefficients, because ULS ensures that the residuals of the estimated covariances sum to zero (Savalei (2014), see also MacCallum et al. (2007)), thereby avoiding bias in the reliability estimates. We consider it an interesting topic for future research to define reliability coefficients for multi‐item composites in SRM designs within the generalizability theory framework (see Ten Hove et al. (2025) for inter‐rater reliability coefficients when social networks of interacting subjects were evaluated by external raters). The multivariate SRM (see Equation 5) can be used to obtain the corresponding variance and covariance parameters. A notable feature of the generalizability theory framework is that different reliability coefficients can be estimated without relying on a factor model (Brennan (2001), see also Tryon (1957)). More specifically, unidimensionality assumptions are not required in the definition of the reliability coefficients in this framework.

Finally, there are other ways how the reliability of a composite can be conceptualized. For example, in case of longitudinal round‐robin data, a measure retest‐reliability might be of interest. In this context, it would also be possible to examine reliability indices based on (longitudinal) state‐trait variance decompositions such as the STARTS model (Kenny & Zautra, 2001; Nestler et al., 2025) or the latent state‐trait model (Eid et al., 2025). We believe that the investigation of such alternative reliability measures could also be interesting for the SRM context. Moreover, in this manuscript we assumed that the round‐robin variables are continuous. It might therefore be interesting to extend the SR‐CFA to categorical round‐robin data and to derive reliability indices for these data as well. Recent research (Cho & Beland, 2025) suggests that ordinal reliability estimators are on average not superior to continuous estimators in the case of ordinal data, and it may be interesting to further investigate this question for the complex dyadic interdependent data considered here.

### 7.2 Relationship to Other SRM Scores

When we introduced the aims of this article we mentioned that–to the best of our knowledge–no scale‐type reliability index has yet been proposed in the SRM literature. However, there are some articles (Bond & Malloy, 2023; Bonito & Kenny, 2010; Lüdtke et al., 2018; Warner et al., 1979) that showed how to quantify the accuracy (or reliability) of the estimated SRM effects (e.g. the estimate of the perceiver effect ${\hat{p}}_{i}$ of person i for the nervousness judgment). In this section, we briefly discuss the relationship between our person‐level composites and these SRM effect scores, because this allows us to highlight another question for future research that we think is important, namely, the derivation of a more general theory for estimating SRM effects.

To simplify the following discussion, we first focus on the case where a single round‐robin variable has been assessed, and we assume that this variable is mean centred. In this case, the sum of the round‐robin judgments for perceiver i is

(35) $$\begin{array}{l} {\hat{p}}_{S,i}=w_{i}^{p}y \end{array}$$

where $w_{i}^{p}$ is a row vector that controls whether a judgment in y is included in the sum 'for' person i. For example, in a three‐person round‐robin group, the sum of the round‐robin judgments for the first person can be calculated with:

(36) $$\begin{array}{l} {\hat{p}}_{S,1}=\left(\begin{array}{cccccc} 1 & 0 & 1 & 0 & 0 & 0 \end{array}\right)y \end{array}$$

where we assumed that y is ordered as ${\left(y_{12},y_{21},y_{13},y_{31},y_{23},y_{32}\right)}^{\prime }$. Thus, estimates are calculated by *weighting* those judgments with one in which the first person is the perceiver while all other judgments are multiplied by zero.

We used ${\hat{p}}_{S,i}$ in Equation 35 because the raw sum score provides an estimate of person i's (latent) perceiver effect. In fact, in the psychometric literature, the sum score is one of the most common approaches for estimating a person's true (or factor) score. In the SRM literature, researchers sometimes use another estimator for the perceiver effect (and also the target effect; see Bonito & Kenny, 2010; Warner et al., 1979):

(37) $$\begin{array}{ll} {\hat{p}}_{BK,i} & =\frac{{\left(n-1\right)}^{2}}{n(n-2)}{\bar{y}}_{i.}+\frac{\left(n-1\right)}{n(n-2)}{\bar{y}}_{.i}=\frac{\left(n-1\right)}{n(n-2)}\underset{j=1,j\neq i}{\sum }y_{ij}+\frac{1}{n(n-2)}\underset{j=1,j\neq i}{\sum }y_{ji}\; \end{array}$$

where the right‐hand side of the equation shows that ${\hat{p}}_{BK,i}$ combines the sum of i's judgment as a perceiver and as target. Importantly, for the first person in a three‐person round‐robin group, Equation 37 can also be written as a weighted sum:

(38) $$\begin{array}{l} {\hat{p}}_{BK,1}=\frac{1}{n(n-2)}\left(\begin{array}{cccccc} \left(n-1\right) & 1 & \left(n-1\right) & 1 & 0 & 0 \end{array}\right)y \end{array}$$

This shows that Bonito and Kenny's approach uses 'only' a different weighting scheme compared to the sum score (e.g. the group size is considered in the weight).

Furthermore, Bond and Malloy (2023) recently suggested to estimate the perceiver and target effects of person i with

(39) $$\begin{array}{l} {\hat{u}}_{i}={\left(\begin{array}{cc} {\hat{p}}_{EAP,i} & {\hat{t}}_{EAP,i} \end{array}\right)}^{\prime }={\hat{\Sigma }}_{u}Z_{i}^{\prime }{\hat{\Sigma }}_{y}^{-1}y \end{array}$$

where ${\hat{u}}_{i}$ are called expected a posteriori estimates in the psychometric literature (EAPs, see Skrondal & Rabe‐Hesketh, 2004) and in the CFA literature these are the (factor score) estimates that are obtained when one uses the regression method (Devlieger et al., 2016; Mulaik, 2009). Equation 39 is also a 'weighting' estimator

(40) $${\hat{p}}_{EAP,1}=e_{1}^{\prime }{\hat{\Sigma }}_{u}Z_{i}^{\prime }{\hat{\Sigma }}_{y}^{-1}y=w_{i}^{p}y$$

where $e_{1}^{\prime }$ is a 2×1 unit vector with the first element being one; we use this vector to select the first row in ${\hat{\Sigma }}_{u}Z_{i}^{\prime }{\hat{\Sigma }}_{y}^{-1}$.

To summarize, all three estimators can be written as a weighted linear combination of the observed round‐robin judgments: while the EAP is a function of estimated model parameters, the other two estimators use fixed weights. Furthermore, since a weighting scheme determines which components of the round‐robin judgments are considered in the estimate, this also determines the reliability of this estimate. For instance, we showed that the reliability of the perceiver effect estimate with the sum score is

(41) $$\begin{array}{ll} \rho_{p_{S}} & =\frac{\sigma_{p}^{2}}{\sigma_{p}^{2}+\sigma_{t}^{2}/(n-1)+\sigma_{r}^{2}/(n-1)}\; \end{array}$$

and Bonito and Kenny (2010) showed (see also, Lüdtke et al., 2018) that the reliability of their estimate is

(42) $$\rho_{p_{BK}}=\frac{\sigma_{p}^{2}}{\sigma_{p}^{2}+\frac{n-1}{n(n-2)}\sigma_{r}^{2}+\frac{1}{n(n-2)}\sigma_{rr}}.$$

Thus, their estimate depends on the size of the group, the relationship variance and the dyadic reciprocity correlation, but is independent of the variance of the target effects because these effects are eliminated when the formula is solved (Lüdtke et al., 2018).

This result can also be derived on a more general level by writing the whole round‐robin judgment vector y as the sum of a true score vector and a measurement error vector

(43) $$\begin{array}{ll} y & =\tau +\epsilon \\ & =\overset{I}{\underset{i=1}{\sum }}Z_{i}\Lambda_{u}\eta_{u_{i}}+\overset{D}{\underset{d=1}{\sum }}W_{d}\Lambda_{r}\eta_{r_{d}}+\overset{I}{\underset{i=1}{\sum }}Z_{i}\epsilon_{u_{i}}+\overset{D}{\underset{d=1}{\sum }}W_{d}\epsilon_{r_{d}}\; \end{array}$$

and by noting that an estimate of the reliability of a person‐level effect can then be obtained with

(44) $$\begin{array}{ll} \rho_{p} & =\frac{w_{i}\left[\sum_{i=1}^{I}Z_{i}\Lambda_{u}\Phi_{u}\Lambda_{u}^{\prime }Z_{i}^{\prime }\right]w_{i}^{\prime }}{w_{i}\Sigma_{y}w_{i}^{\prime }}\; \end{array}$$

This equation is important as it would allow for obtaining a reliability estimate for any weighting scheme. We consider these considerations interesting because we believe that they allow to derive a more general theory for estimating SRM effects and their reliabilities. Ideally, such a theory would allow for a more informed selection of the 'correct' SRM effect in a certain empirical application, and thus further strengthen the analyses of round‐robin data.

### 7.3 Conclusion

The present article introduced a general approach for estimating the reliability of composites of round‐robin variables using the SR‐CFA. We derived formulas for calculating reliability coefficients for both dyad‐level and person‐level composites, which can accommodate various factor structures and measurement restrictions. The proposed method allows researchers to quantify measurement error in round‐robin composites, thereby providing essential information for evaluating observed effects. We hope that this will strengthen the quality and interpretability of round‐robin research across diverse areas of psychological science.

### AUTHOR CONTRIBUTIONS

**Steffen Nestler:** conceptualization; writing – original draft; methodology; writing – review and editing; formal analysis. **Oliver Lüdtke:** conceptualization; writing – original draft; writing – review and editing. **Alexander Robitzsch:** conceptualization; writing – original draft; software; methodology.

## CONFLICTS OF INTEREST

The authors declare no conflicts of interest.

## AIGC TOOL STATEMENT

AI tools were used for checking grammar and language in this article.

## Data Availability

We share the data and R code for the illustrative data example publicly on the Open Science Framework (https://osf.io/tde49/?view_only=1a67a1b305fd4f64980ced1bd1ca06e2). The data that support the findings of this study are openly available in Open Science Forum at https://osf.io/tde49/?view_only=1a67a1b305fd4f64980ced1bd1ca06e2.

## Footnotes

^1^
The error decomposition in Equation (17) follows from assuming a factor model for each SRM effect (i.e. each SRM effect is a linear function of the factors plus a component unexplained by the factors). Alternative definitions of the error are possible, for instance, in terms of stochastic measurement theory (Eid, 2000). We note that such alternative definitions can be easily handled in the present framework by imposing certain parameter constraints on the residual covariance matrices.

^2^
As we explain in the discussion, the unit vector 1 can be replaced by a vector with unequal weights.
